# Detection of Early Signs of Diabetic Retinopathy Based on Textural and Morphological Information in Fundus Images

**DOI:** 10.3390/s20041005

**Published:** 2020-02-13

**Authors:** Adrián Colomer, Jorge Igual, Valery Naranjo

**Affiliations:** 1 Institute of Research and Innovation in Bioengineering, I3B, Universitat Politècnica de València, 46022 Valencia, Spain; adcogra@i3b.upv.es (A.C.); vnaranjo@dcom.upv.es (V.N.); 2 Departamento de Comunicaciones, ITEAM Research Institute, Universitat Politècnica de València, 46022 Valencia, Spain

**Keywords:** biomedical image processing, diabetic retinopathy, classification, granulometry-based descriptor, LBP, hand-driven learning, exudates, microaneurysms

## Abstract

Estimated blind people in the world will exceed 40 million by 2025. To develop novel algorithms based on fundus image descriptors that allow the automatic classification of retinal tissue into healthy and pathological in early stages is necessary. In this paper, we focus on one of the most common pathologies in the current society: diabetic retinopathy. The proposed method avoids the necessity of lesion segmentation or candidate map generation before the classification stage. Local binary patterns and granulometric profiles are locally computed to extract texture and morphological information from retinal images. Different combinations of this information feed classification algorithms to optimally discriminate bright and dark lesions from healthy tissues. Through several experiments, the ability of the proposed system to identify diabetic retinopathy signs is validated using different public databases with a large degree of variability and without image exclusion.

## 1. Introduction

At least 2.2 billion people around the world have a vision impairment, of whom at least 1 billion have a vision impairment that could have been prevented or is yet to be addressed [1]. Eye conditions such as cataracts, trachoma and refractive error are the main focus of eye care strategies. The combination of a growing and ageing population will significantly increase the total number of people with eye conditions and vision impairment (estimated blind people in the world will exceed 40 million by 2025). Other common eye conditions reported by the World Health Organization include: myopia (near-sightedness), late detection in poorly integrated eye care services, and diabetic retinopathy (increasing numbers of people are living with diabetes, particularly Type 2; nearly all people with diabetes will have some form of retinopathy in their lifetimes) [1]. For this reason, the early detection of diabetic retinopathy is essential to guarantee the maintenance of the vision. The first signs of diabetic retinopathy can be noticed using fundus photographs acquired by means of a retinal camera.

Diabetes occurs when the pancreas does not secrete enough insulin, or the body is unable to process it properly. Diabetes affects the circulatory system, and therefore to the retina. When fluid leaks from blood vessels into the retina, this is damaged and this medical condition is called diabetic retinopathy (DR) [2,3].

**Figure 1:**
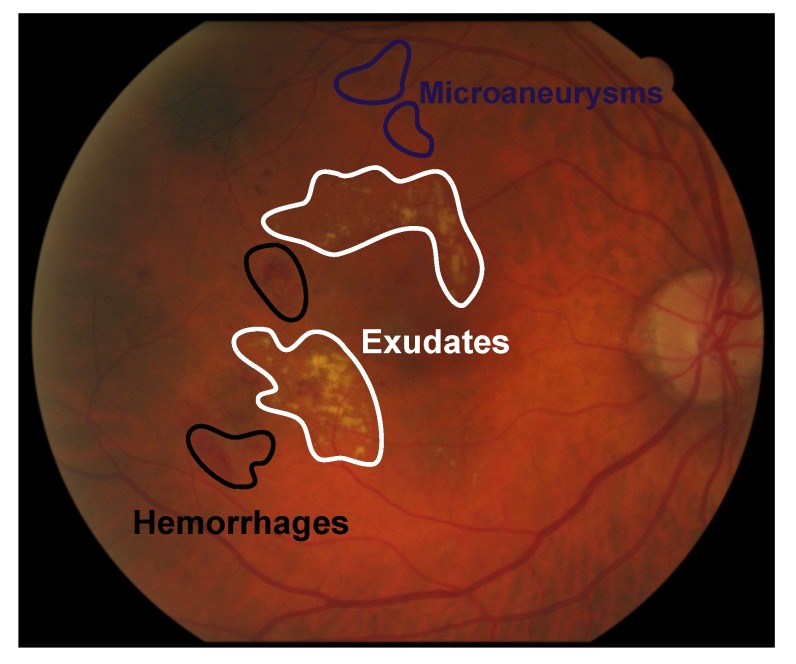
Example of exudates, microaneurysms and hemorrhages in fundus images.

For automatic screening of diabetic retinopathy through fundus imaging, systems are based on the detection of specific anomalous patterns or lesions [4], e.g., microaneurysms (small saccular dilations of capillaries that appears as round spots of dark red color with sharp edges in the retina background), exudates (deposits of lipids and proteins in the retina that produce bright lesions of yellowish-white color with prominent and irregular edges), cotton wool spot (a result of accumulations of axoplasmic material within the nerve fiber layer that appear as fluffy white patches on the retina) and hemorrhages (wide accumulation of blood in the retina). In this paper, we focus on the detection of all these lesions because they are the earliest visible signs of the disease. The goal is to distinguish healthy from pathological areas of the image considering both cases bright and dark retinal damage and proposing a unique feature vector able to encode the relevant information in the two cases. We show an example of the most common DR signs in Figure 1.

Depending on the evolution with time, diabetic retinopathy is classified as Non-Proliferative and Proliferative [5]. Non-Proliferative is the earliest stage of the pathology and it is characterized by deposits of extra fluid and small amounts of blood, from the vessels into the retina, exudates and microaneurysms. It usually progresses to develop the Proliferative one because many blood vessels in the retina are closed hindering the proper blood flow. The retina responses to this fact by growing new abnormal blood vessels (this process is called neovascularization).

Routine eye checks and good diabetes control can protect people’s vision from this condition. The action plan presented by the WHO [6] reflects the eye care services need to become an integral and established the screening campaigns, as key method for detecting retinal pathologies in their early stage [6]. However, these proposals require a large workload of trained experts in the identification of anomalous retinal patterns. This fact along with the increasing of the population at risk, make these proposals economically unfeasible. Moreover, it must be pointed out that this type of retinal disease diagnosis is highly influenced by the inherent subjectivity of each expert. These arguments suggest the need for developing automatic screening systems that can be used in primary health care reducing the working time to the specialists and providing the location and quantification of the pathological retinal tissue propitiated by retinal diseases.

Automatic screening systems based on digital imaging should be supported by acquisition devices of high resolution to guarantee the successful of the diagnosis in difficult cases. Algorithms able to extract the relevant information from fundus images have become increasingly important for the automatic screening and to provide clinicians support in the diagnosis phase.

There are two machine learning paradigms to try to solve the problem: the traditional classification approach where the input is a feature vector obtained from the fundus images [7,8,9], and the deep learning approach (in particular, using convolutional neural networks) [10,11,12]. While the second approach usually gets better classification results, most of these methods do not provide understandable interpretations about the relevant features of the different pathological signs in the retina, so its clinical usefulness is questionable until more research efforts in the interpretation of the high-level features extracted by the convolutional blocks of a CNN will be done. It is unlikely that a black box classification system is going to be accepted by any ophthalmologist in the real world, no matter the good the results are in previous experiments. Another important issue is the limited amount of labeled fundus images required by deep learning strategies. Some recent works are trying to overcome these limitations by creating synthetic data [13,14,15].

The approach presented in this paper belongs to the traditional classification approach. In that case, the most common procedure is to segment these lesions using different methods [16,17,18]. These approaches present a high false-positive rate at pixel level. This fact is the main motivation of the new perspective proposed in this work, in which the characterization of healthy and damaged retinal areas is studied by applying image descriptors in a local way, avoiding the segmentation step. Our approach is different to previous works based on feature extraction and classification. In [19] a previous segmentation of exudates is required to perform the feature extraction. The most habitual procedure is to extract features from a lesion candidate map generated by different techniques such as mathematical morphology [16,20,21], background subtraction [22], clustering [23] or using banks of filters and applying a low adaptive threshold [24,25] among others. To the best of the authors’ knowledge, the work proposed by Quellec et al. [26] is the unique previous attempt which has some resemblance with the strategy proposed in this paper. The main difference is that they use a local analysis without overlapping based on wavelet features; area under the receiver operating curve 0.761 was reported in e-ophtha public database. In our paper, robust descriptors extract texture, shape and roughness features from the visual information of retinal images. This methodology does not require the previous segmentation of retinal lesions or the generation of candidate maps, avoiding error sources due to the segmentation process and computational cost.

## 2. Materials

Two public fundus databases were used to validate the methods proposed throughout this paper.

- E-OPHTHA [27] is a database of fundus images especially designed for diabetic retinopathy screening. This public database is divided in two subsets depending on the lesion type: exudates and microaneurysms. These lesions are manually annotated by experts and the ground truth is provided. In this paper, we will use the exudates subset (*E-OPHTHA_EX*) that is composed by 47 pathological images (Pathological_EX) and 35 images with no lesion (Healthy_EX). All the retinal images were acquired with the same field of view angle 40°, and they present different spatial resolutions: 13 images with 1440×960 pixels, 2 with 1504×1000, 9 with 2048×1360 and 23 with the highest resolution 2544×1696).
- DIARETDB1 public database [28] consists of 89 color fundus images, of which 84 contain at least mild non-proliferative signs of diabetic retinopathy and five are considered to be normal by medical experts. In particular, 41 fundus images show bright lesions (exudates) and 45 contain dark lesions (microaneurysms or hemorrhages). The angle of vision is 50° and resolution 1500×1152 pixels. The ground truth used in this paper is the one proposed by the authors of the database (when 3 out of 4 experts label the pixel as pathological, it is considered to be an exudate pixel).

### 2.1. Data Conditioning

The original fundus images must be preprocessed to take into account three issues that can influence the performance of the classifiers: image resolution, color and the presence of blood vessels.

- Resolution normalization
  The E-OPHTHA_EX database is composed by images with four different resolutions. The images are resized to the dimensions of the smallest image (1440×960) after a local maximum filter is applied in order to preserve the small bright lesions [29] (complementary a minimum filter is used to keep microaneurysms).
- Color normalization
  In fundus images, the green component of the RGB representation shows the maximum contrast between lesions and background. The red channel is often saturated and has low contrast, and the blue channel is very noisy and suffers poor dynamic range. For these reasons, the green component is commonly used to segment the lesions [16,20,30] and it is used in this work. However, the green mean value differs significantly for different images even in the same database due to incorrect white balance. To normalize images, we apply a color transformation with the aim of increasing their color homogeneity [31].
- Impainting blood vessels
  Blood vessels cover a high percentage of the fundus image hindering the automatic detection of important structures as optic disc, optic cup and macula, among others. Vessels are also considered to be noise or artefacts that hamper the segmentation of different lesions such as exudates, microaneurysms and drusen among others and also the classification of pathologies based on background textures. Retinal vessels segmentation techniques aim to separate the different retinal vasculature structure tissues from the fundus image background and aforementioned retinal anatomical structures such as optic disc, macula, and abnormal lesions. See [32] for a review of retinal vessels segmentation algorithms, and [33] for a review of optic disc segmentation for glaucoma detection.
  A possible procedure to avoid blood vessels is to consider these structures as missing pixels and trying to restore them using the background. Image inpainting is a technique for restoring missing or damaged areas in a digital image [34]. Inpainting methods assume that pixels in the known and unknown areas of the image have the same statistical properties or geometrical structures. Different algorithms exist in the literature [35]; we will use a sparse-based inpainting method with spread neighborhoods specifically designed to inpaint blood vessels in fundus images [36].

### 2.2. Image Patches: Input Features and Class Labels

**Figure 2:**
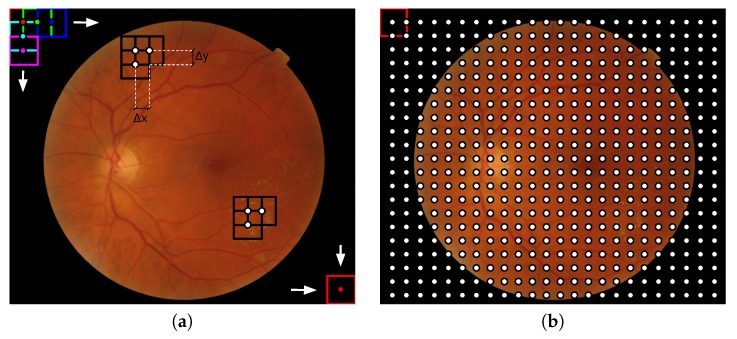
Illustration of the local analysis in fundus images: (**a**) Visual representation of how the sliding window of dimensions $N_{w}\times N_{w}$ loops a fundus image and (**b**) the resulting image grid composed by the centers of the sliding window in each position. This process is equivalent to a dense sampling. For the representation $N_{w}=64$ and (Δx,Δy) = (32,32) were used.

The lesions induced by diabetic retinopathy present different sizes according to the stage of the disease. In the feature extraction stage of the proposed methodology, descriptors are locally calculated: the image is divided in patches using a sliding window of size $N_{w}\times N_{w}$ and overlap of (Δx,Δy), and image descriptors are computed for each patch. For example, we show the case $N_{w}=64$ and 50% overlapping ($\Delta x=\Delta y=N_{w}/2$) in Figure 2a. This procedure can be related to the way in which the human inspects and analyzes an image. From an image-processing point of view, the sliding window strategy is the same as a 2D grid or a dense sampling of the image, in which the samples correspond to the central pixel of the window. Figure 2 represents the whole procedure and equivalent dense image sampling.

**Figure 3:**
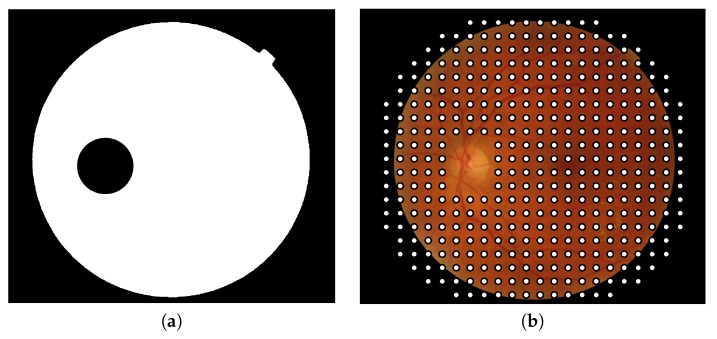
(**a**) Binary mask used to exclude the patches containing optic disk pixels and the patches located out of the field of view and (**b**) center of the patches composing the final grid in which image descriptors will be applied.

It is important to note that patches should contain at least one pixel belonging to retinal texture to be processed; in other words, patches contained entirely outside the lens are discarded. In addition, patches containing optic disk pixels, obtained by the method proposed in [37], are not considered in the process (Figure 3).

## 3. Methods

In this section, we present the proposed image descriptors (features) and classifiers used to distinguish between the healthy and pathological retinal tissue. The features are obtained from the patches obtained in the previous section. We explore different descriptors: local binary patterns to encode the texture of pathological patches and hierarchical morphological operators to encode granularity. We will use these features as the input to the classifier.

### 3.1. Local Binary Pattern Variance

Local Binary Patterns (LBP) is a powerful feature for texture classification [38]. LBP establishes a label for each pixel taking into account its neighborhood which is defined by a radius *R* and number of points *P*:

(1) $$LBP_{P,R}\left(i,j\right)=\sum_{p=0}^{P-1}s\left(g_{p}-g_{c}\right)\cdot 2^{p},\;s\left(x\right)=\left\{\begin{array}{cc} 1 & ifx\geq 0\\ 0 & ifx<0 \end{array}\right.$$

where *P* represents the number of samples on the symmetric circular neighborhood of radious *R*, $g_{c}$ is the gray value of pixel (i,j) and $g_{p}$ the gray value of each neighbor. The final $LBP_{P,R}$ label is obtained by converting the binary string into a decimal value. $LBP_{P,R}$ texture operator describes the occurrence of specific patterns in the neighborhood of each pixel in a P-dimensional histogram.

LBP technique has been previously used in fundus images mostly for the segmentation of retinal vessel [39,40] and the automatic identification of ophthalmic diseases [9,19,41]. We will use the rotation-invariant uniform LBP implementation $LBP_{P,R}^{ruin}$ [42]. Using this LBP variant, with a level of uniformity of U=2 and P=8, ten different texture labels could be generated depending on the binary string computed in the comparison between a pixel and its neighborhood.

**Figure 4:**
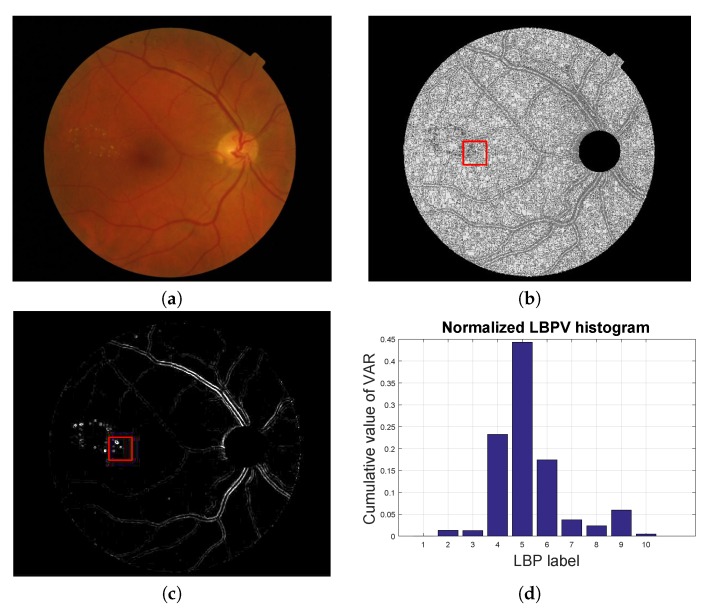
Local texture analysis. (**a**) Original fundus image, (**b**) LBP image and (**c**) VAR image with a patch highlighted in red and (**d**) the LBPV normalized histogram computed for the red patch.

To boost the performance of LBP and to obtain a texture descriptor invariant against shifts in gray scale, the complementary measure $VAR_{P,R}$ is also computed and combined with the $LBP_{P,R}^{riu2}$ obtaining the final feature vector $LBPV_{P,R}$ as follows:

(2) $$VAR_{P,R}\left(i,j\right)=\frac{1}{P}\sum_{p=0}^{P-1}{\left(g_{p}-\mu \right)}^{2},\;\mu =\frac{1}{P}\sum_{p=0}^{P-1}g_{p}$$

(3) $$LBPV_{P,R}\left(k\right)=\sum_{i=1}^{M_{1}}\sum_{j=1}^{M_{2}}w\left(LBP_{P,R}\left(i,j\right),k\right),\;k\in \left[0,K\right]$$

where:

(4) $$w\left(LBP_{P,R}\left(i,j\right),k\right)=\left\{\begin{array}{cc} VAR_{P,R}\left(i,j\right), & LBP_{P,R}\left(i,j\right)=k\\ 0, & otherwise. \end{array}\right.$$

where *K* is the maximal LBP label. In Figure 4 we show a graphical example of how this feature vector is obtained for a given patch.

### 3.2. Ganulometric Profile

One of the most interesting techniques based on mathematical morphology is granulometry [43,44]. In [45] this technique is used with the aim of detecting neovascularization, i.e., the abnormal formation of blood vessels in the retina due to the lack of oxygen.

Given a gray-level image f∈F(E,T), its dilation (erosion) by a flat structuring element *B* is introduced as the dilation (erosion) of each level set $X_{t}\left(f\right)$ by *B*:

(5) $$\begin{array}{c} \begin{array}{c} \delta_{B}\left(f\right)\left(x\right)=sup\left\{t_{l}|x\in \delta_{B}\left(X_{t_{l}}\right)\right\},\;t_{l}\in T\\ \epsilon_{B}\left(f\right)\left(x\right)=inf\left\{t_{l}|x\in \epsilon_{B}\left(X_{t_{l}}\right)\right\},\;t_{l}\in T \end{array} \end{array}$$

The two elementary operations of gray-level erosion and dilation can be composed together to yield a new set of gray-level operators with desirable feature extractor properties which are given by, the gray-level opening:

(6) $$\gamma_{B}\left(f\right)\left(x\right)=\left(f\circ B\right)\left(x\right)=\delta_{B}\left(\epsilon_{B}\left(f\right)\right)\left(x\right)$$

and the gray-level closing:

(7) $$\varphi_{B}\left(f\right)\left(x\right)=\left(f•B\right)\left(x\right)=\epsilon_{B}\left(\delta_{B}\left(f\right)\right)\left(x\right)$$

Making use of the previously explained operators, a shape descriptor can be defined. Let us first consider an opening $\gamma_{i}\left(f\right)$, applied to an image *f* with a structuring element (SE) of size *i*. The opening can be computed as a sequence of an erosion followed by a dilation. When opening is computed on the image with a SE of increasing size (λ), we obtain a morphological opening pyramid (or granulometry profile) which can be formalized as:

(8) $$\Pi_{\gamma }\left(f\right)=\left\{\Pi_{\gamma \lambda }:\Pi_{\gamma \lambda }=\gamma_{\lambda }\left(f\right),\forall \lambda \in \left[0,\ldots ,n_{max}\right]\right\}$$

where $n_{max}$ represents the maximum size of the structuring element.

By duality, a closing, $\varphi_{i}\left(f\right)$ is defined as the dilation of an original image *f* with a SE of size *i*. In the same way, a morphological closing pyramid is an anti-granulometry profile and can be computed on the image performing repeated closings with a SE of increasing size (λ) defined as:

(9) $$\Pi_{\varphi }\left(f\right)=\left\{\Pi_{\varphi \lambda }:\Pi_{\varphi_{\lambda }}=\varphi_{\lambda }\left(f\right),\forall \lambda \in \left[0,\ldots ,n_{max}\right]\right\}$$

**Figure 5:**
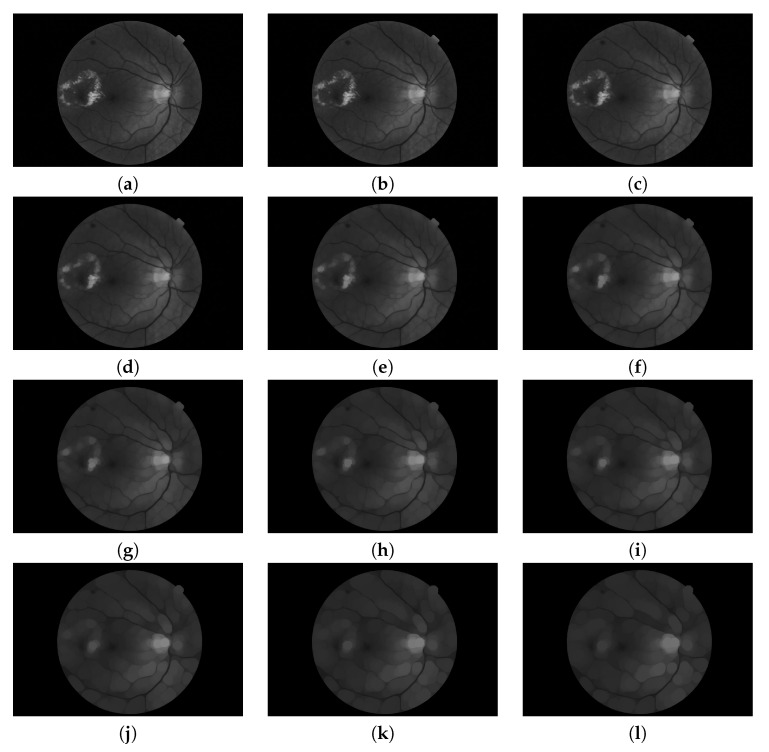
Pyramid of openings computed for a fundus image using an isotropic structuring element. It is composed by 12 images (n=0,2,4,…,22). (**a**) Original image; i.e., $\gamma_{0}$ is the identity mapping; (**b–k**) are the results of applying the opening operator with increasing size (according to s=2) and (**l**) is the last image of the pyramid corresponding to the operation $\gamma_{nmax}$.

**Figure 6:**
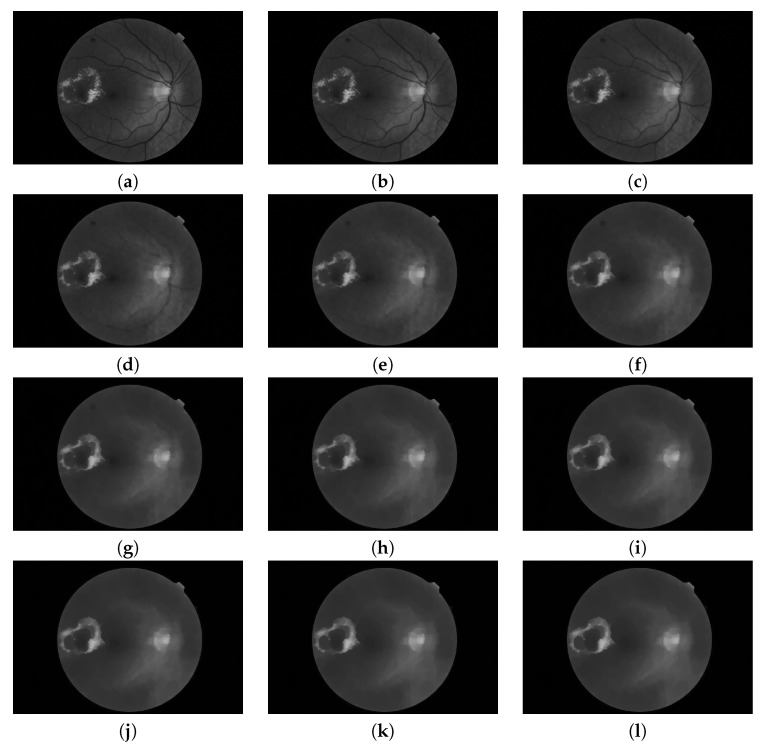
Pyramid of closings computed from a fundus image using an isotropic structuring element. It is composed by 12 images (n=0,2,4,…,22). (**a**) Original image; i.e., $\varphi_{0}$ is the identity mapping; (**b–k**) are the results of applying the closing operator with increasing size (according to s=2) and (**l**) is the last image of the pyramid corresponding to the operation $\varphi_{nmax}$.

In Figure 5 and Figure 6 we show the different levels of the pyramids $\Pi_{\gamma }$ and $\Pi_{\varphi }$ for n=0,2,4,…,22 for a particular fundus image, respectively.

Let m(f) be the Lebesge measure of a discrete image *f*: m(f) is the area of *f* in the binary case (number of pixels) and the volume in the grey-scale case (sum of pixel values). Making use of the morphological pyramids established above, a shape descriptor can be defined. The granulometry curve, or pattern spectrum of *f* with respect to Γ is defined as the following (normalized) mapping:

(10) $$PS_{\Gamma }\left(f,n\right)=PS\left(f,n\right)=\frac{m\left(\Pi_{\gamma n}\left(f\right)\right)-m\left(\Pi_{\gamma n+1}\left(f\right)\right)}{m(f)},\;n\geq 0$$

The pattern spectrum $PS_{\Gamma }\left(f,n\right)$ (also called size density of *f*) maps each size *n* to some measure of the bright image structures with this size: loss of bright image structures between two successive openings. It is a probability density function (a histogram) in which a large impulse in the pattern spectrum at a given scale indicates the presence of many image structures at that scale.

By duality, the concept of pattern spectrum extends to anti-granulometry curve $PS_{\Phi }\left(f\right)$, by closings $\varphi_{n}\left(f\right)$, stacked in the morphological pyramid $\Pi_{\varphi }$:

(11) $$PS_{\Phi }\left(f,-n\right)=PS\left(f,-n\right)=\frac{m\left(\Pi_{\varphi n}\left(f\right)\right)-m\left(\Pi_{\varphi n-1}\left(f\right)\right)}{m(f)},\;n\geq 0$$

This spectrum characterizes the size of dark image structures. Both granulometry curve and anti-granulometry curve can be appended into a unique curve, with closings versus size on the left side (negative side) and openings versus size on the right side (positive side) of the diagram; i.e.:

(12) $$\left\{-n,0,n\right\}\to PS\left(f,n\right)=\{PS_{\Phi }\left(f,-n\right),0,PS_{\Gamma }\left(f,n\right)\},\;n\geq 0$$

**Figure 7:**
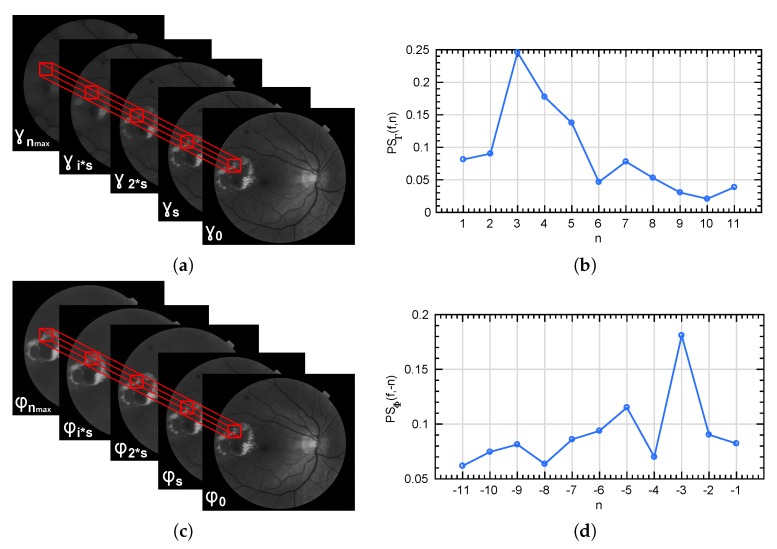
(**a**,**c**) represent the local extraction of the granulometry and anti-granulometry curves from the morphological pyramids $\Pi_{\gamma }$ and $\Pi_{\varphi }$. (**b**,**d**) show the pattern spectrum computed for the patch marked in red in each case.

**Figure 8:**
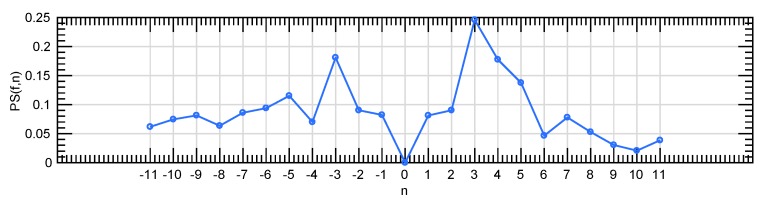
A global pattern spectrum extracted by the combination of the granulometric and anti-granulometric profiles.

From the morphological pyramids, a local description of the shape and size of the retinal texture is performed by computing the pattern spectrum of squared patches. In particular, $PS_{\Gamma }^{⋄}\left(f,n\right)$ and $PS_{\Phi }^{⋄}\left(f,n\right)$ are locally computed for each patch of the green channel of fundus images (extracted according to the explanation in Section 2.2), following Equations (10) and (11) respectively. In addition, the combination of both descriptors takes place to the curve $PS^{⋄}\left(f,n\right)$ according to Equation (12). Please note that the ⋄ symbol denotes the locality of the descriptor. In Figure 7a,c, the local extraction of the granulometry and anti-granulometry curves from the morphological pyramids $\Pi_{\gamma }$ and $\Pi_{\varphi }$ is represented. $PS_{\Gamma }^{⋄}\left(f,n\right)$ and $PS_{\Phi }^{⋄}\left(f,n\right)$ descriptors extracted from the patch marked in red in a fundus image are reported in Figure 7b,d. In addition, the whole pattern spectrum $PS^{⋄}\left(f,n\right)$ is shown in Figure 8.

### 3.3. Classifiers

We tested three different classifiers: random forests (RF), support vector machines (SVM) and Gaussian processes for classification (GPCs). RF was chosen as the most used tree-structured predictor in the literature to address classification problems. One of its major strengths is the low computational time required in the training stage while its principal disadvantage is that RF usually suffer from overfitting when using high-dimensional feature vectors. To make face this problem, a kernel-based method was additionally selected as robust method against overfitting, i.e., SVM with Radial Basis Function (RBF) kernel. Finally, to solve the problem under study from other point of view, a probabilistic classification algorithm was also involved in the experimental stage, i.e., GPCs.

- Random Forests are a combination of tree-structured predictors $\left\{h\left(x,\Theta_{k}\right),k=1,\ldots ,K\right\}$ so that each tree depends on the training set and values of a random vector, $\left\{\Theta_{k}\right\}$, independently sampled of the past random vectors $\left\{\Theta_{1},\ldots ,\Theta_{k-1}\right\}$ and with the same distribution for all trees in the forest [46]. To predict a new instance, it is pushed down the tree. Then, the label assigned to the instance will be the label corresponding to the terminal node of the tree. This process is iterated by the *K* predictors or trees and the final classification label will be obtained by majority voting of the trees.
- Support Vector Machine (SVM) builds a hyperplane of separation in the input space maximizing the distance or margin with respect to the supported vectors of the different classes [47,48]. The input data is usually kernelized in order to take into account classes non-linearly separable. We will use the radial basis function (RBF) kernel $K\left(x_{i},x_{j}\right)=e^{-\gamma ∥x_{i}-x_{j}{∥}^{2}},\gamma >0$. Classification experiments using a linear kernel are also performed to establish a baseline method for comparison purposes.
- Gaussian processes for classification. Random forests and SVM are examples of discriminative classifiers (they look for the decision boundary between the classes). A generative classifier obtains a probabilistic model of the classes that is used for future classification purposes maximizing the posterior probability. As an example of a probabilistic classification method, we use Gaussian processes for classification purposes (GPCs) [49].

### 3.4. Hand-Driven Learning Procedure

The 47 images of *E-OPHTHA exudates* database were divided in K=5 partitions. External cross-validation, using the “leave-one-out” technique, allowed us to carry out a fair validation of the proposed method. During preprocessing, the images are resized so all of them have the same angle of view and color transformation and image inpainting procedures are carried out. After the preprocessing, the green component is extracted, the patches are obtained and the descriptors for each patch are calculated. The size of the patches is 64×64 pixels and the displacement between patches is (Δx,Δy) = (32,32) pixels.

**Figure 9:**
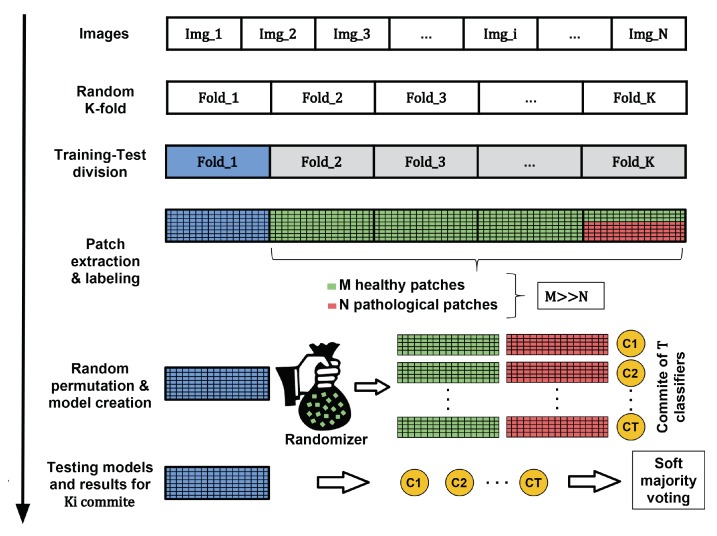
Process of creating the machine learning models using a generic dataset. Green and red samples are used in the creation of the model while blue instances refer to the samples used in the testing stage.

Since pathological areas represent only small regions of the whole retinal image (less than one percent of the total number of pixels that compose the retinal image in most cases), the patch extraction process and corresponding feature vector extraction will result in an imbalanced dataset. Training a classifier with an imbalanced dataset can produce overfitting to the majority class (“healthy” in our case) [50]. To avoid this problem, we proceed as follows. Let us assume that the number of healthy and pathological samples are *M* and *N* respectively where M>>N. Thus, the set of all healthy samples is randomly permuted and partitioned into $T=round\left(M/N\right)$ subsets with the same cardinality as the number of pathological samples. A committee of *T* classifiers is then learned with training sets formed by joining all pathological training samples and each partition of healthy training samples. In the test stage, testing samples are evaluated for each of the *T* models and soft majority voting is applied to the output probabilities as final criterion. If the obtained probability is higher than a given threshold δ (typically δ=0.5), the patch is assigned to the class “pathological”. An overview of the whole process can be observed in Figure 9. In our case, T=9 classifiers compose the decision committee to classify each testing instance.

## 4. Results

### 4.1. Performance Measures

Common metrics in the field of machine learning are used to evaluate the classification performance in each experiment.

Accuracy is determined by the sum of correct predictions divided by the total number of cases. It shows how correct a diagnostic test identifies and excludes a given condition.

(13) $$Accuracy=\frac{TP+TN}{TP+TN+FP+FN}$$

where True Positives (TP) and True Negatives (TN) are the correctly predicted values while False Positives (FP) and False Negatives (FN) are the mistakes.

Sensitivity or Recall and Specificity measure the proportion of positive and negative cases which are correctly identified as such, respectively.

(14) $$Sensitivity=\frac{TP}{TP+FN}$$

(15) $$Specificity=\frac{TN}{TN+FP}$$

The receiver operating curve or ROC curve is a graphical plot that illustrates the diagnostic ability of a binary classifier system as its discrimination threshold is varied. The ROC curve is created by plotting the Sensitivity against the 1-Specificity at various threshold settings. The enclosing area of this curve is known as area under ROC curve (AUC) and it is an extended way to measure the predictive modelling accuracy [51]. This measure allows the establishment of fair comparisons where there is a strong imbalance between classes [52]. Based on [53], a diagnostic test in the medical field with an AUC value of 0.5 suggest no discrimination. If the AUC value ranges from 0.7 to 0.8 the test is considered acceptable, if the test presents an AUC value included in the interval 0.8-0.9 it is considered excellent, and more than 0.9 is considered outstanding.

### 4.2. Analysis of Descriptors

In a first set of tests, the behavior of the morphological descriptors to discriminate bright lesions is assessed. For this purpose, E-OPHTHA exudates database was used and four morphological pyramids were computed from the green component of each preprocessed image: two pyramids of openings using both an isotropic ($\Pi_{\gamma_{B}}$) and an angular ($\Pi_{\gamma_{L}}$) structuring element and analogously two pyramids of closings ($\Pi_{\varphi_{B}}$, $\Pi_{\varphi_{L}}$). These morphological pyramids were calculated using an increasing SE size defined by a step (s=2) and a maximum value ($n_{max}=22$). These parameters were optimized making use of the ground truth of the training image dataset. Please note that angular granulometry was computed in directions $0^{\circ }$, $45^{\circ }$, $90^{\circ }$ and $135^{\circ }$. From the morphological pyramids, a local description of the shape and size of the retinal texture was performed by computing the pattern spectrum of squared patches ($PS^{⋄}$) as explained previously (see Figure 7). The classification results obtained for the linear-SVM classifier with different configurations of the feature vector are reported in Table 1. For simplicity in the notation, the $PS^{⋄}$ symbol has been omitted. Please note that feature vectors composed by more than one operator are generated by concatenation of their pattern spectra.

As can be interpreted from the results, when the feature vector is composed by a single pattern spectrum, isotropic granulometries detect better the bright lesion than the directional ones. When a combination of two granulometric patterns is used to feed the SVM classifier, an improvement in the performance classification can be observed, especially when the granulometry and anti-granulometry curves using an isotropic SE are collated in a unique feature vector. The best description of the bright lesions taking into account only size and shape local information is given when all morphological descriptors are used ($\gamma_{B}\gamma_{L}\varphi_{B}\varphi_{L}$). Accuracy and specificity values higher than 76%, a sensitivity of 66% and an AUC value around 0.8 are reported as best classification results in Table 1. These results suggest the need for extracting another kind of information able to support and strengthen the performance of the morphological descriptors.

We test the ability of feature based on local binary patterns LBPV to capture the texture information of healthy and pathological tissues. The parameters are: radius R=1 and number of points in the LBP feature vector P=8, i.e., the dimension of the LBP feature vector is 10 (P+2). Several tests combining shape and texture descriptors were carried out and the obtained results can be read in Table 2.

As we can see, texture information is key in the ability of discriminating between regions containing exudates and non-damaged areas. Moreover, the best classification results are obtained when texture and shape/size information are combined. Improvements around 10% in all the evaluation metrics are registered in comparison with the previous test in which only morphological information was taken into account.

### 4.3. Performance of Different Classifiers

The third experiment aims to analyze if results differ when using different classifiers for the most discriminative feature vector (texture and morphological information).

The *K*-fold procedure and the random permutation for balancing the data (see the procedure represented in Figure 9) were performed using a fix seed to allow fair comparisons among the different classification methods. Table 3 shows the results related to the classification performance of each classifier evaluated on the five external folds of the E-OPHTHA_EX database.

**Figure 10:**
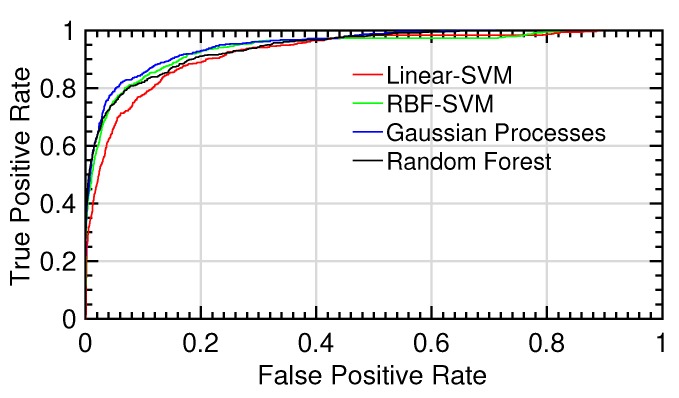
ROC curves for the different tests.ROC curves for the different tests

As it can be observed in that table, different machine learning algorithms maximize specific evaluation metrics, making difficult an overall analysis of their behavior. AUC values provide information about the global performance of each classifier because the ROC curve is calculated by evaluating a sweep of decision thresholds ranging from zero to one (see Figure 10). However, in the medical field, sensitivity and specificity are key parameters for measuring the goodness of proposed methods. For this reason, from the ROC curve of each fold, an optimal decision threshold δ for each ML method was obtained according to the best trade-off between sensitivity and specificity. The same procedure used to optimize the meta-parameters involved in the feature extraction and classification stages was used to optimize the threshold δ. In particular, it was optimized by using the validation dataset for each partition of the external *K*-fold cross-validation. The obtained results can be observed in Table 4.

To evaluate the robustness of the proposed approach, our bright-lesion detection model trained on E-OPHTHA_EX database was used to predict the 47 images containing exudates of *DIARETDB1* database. This test allowed us to perform a deep validation of the presented approach and to establish an exhaustive comparison with the state-of-the-art works related to the automatic exudate detection. Table 5 shows the obtained results using a decision threshold δ=0.5 while Table 6 shows the results maximizing the trade-off between sensitivity and specificity by searching the optimal decision threshold δ for each classification method.

Table 7 compares the exudate detection results obtained in this paper with other works in the same problem, where PPV (positive predicted values) measure the proportion of positive correct predictions with respect to the total number of positive predictions determined by the classification system. Results must be analyzed with care, especially when only some metrics results are included in some papers. The importance of the trade-off between sensitivity and specificity is well-known in the computer-aid diagnostic systems development. In fact, is more convenient to register a higher sensitivity than specificity because the consequences of diagnosing a pathological patient as healthy can be very damaging. As Table 7 shows, the proposed methodology registers the best trade-off between the aforementioned figures of merit. The sensitivity of Walter et al. [16],Welfer et al. [17] and Ghafourian and Pourreza [18] are outperformed by the sensitivity value reported from our system. The only work which makes face our results of exudate detection is Sopharak et al. [21]. However, it is important to highlight that the authors of Sopharak et al. [21] proposed a set of optimally adjusted morphological operators to be used exclusively for exudate detection. In contrast, our method can identify exudates, microaneurysms and hemorrhages using the same feature vector (i.e., encoding textural and morphological information).

**Figure 11:**
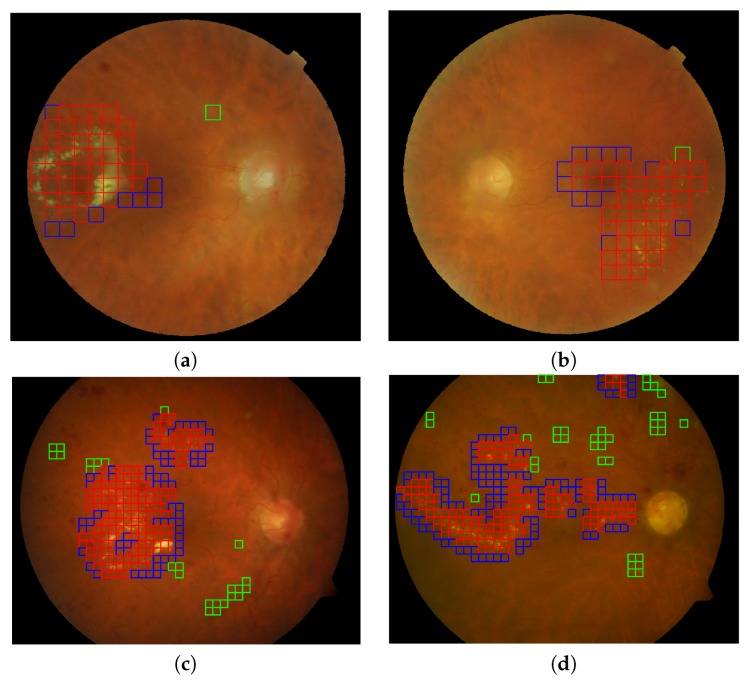
Automatic lesion detection in four retinal images using the best configuration of feature vector and gaussian processes for classification. (**a**,**b**) Two representative images (DS000U30.jpg and DS0009NU.jpg) from the E-OPHTHA exudates database and (**c**,**d**) two representative image (image014.png and image016.png) from DIARETDB1 database. Red squares indicate the true positives, green squares the false positives and the blue squares reveal the false negative detections.

We show a visual example of the result of the classification process in Figure 11. Two images correspond to the E-OPHTHA exudates database and two to the DIARETDB1 database (note that the patches for the DIARETDB1 images are smaller since they have higher resolution). Red patches are detected pathological patches; green ones are healthy patches misclassified as pathological, and blue the pathological patches that were not detected. The rest of the patches not shown in the figure correspond to healthy patches labeled correctly as healthy ones.

### 4.4. Generalization Ability of the Proposed Feature Vector: Microaneurysms and Hemorrhages Detection

In the same way, the proposed feature vector and the classification methods studied in the previous section were used to discriminate between healthy and dark-damaged areas (i.e., patches containing hemorrhages and/or microaneurysms). The material employed to train and validate these models were the 45 images of DIARETDB1 due to the lack of enough dark lesions in the E-OPHTHA database. Table 8 reports the obtained results using a decision threshold δ=0.5 while Table 9 shows the results maximizing the trade-off between sensitivity and specificity by searching the optimal decision threshold δ for each classification method.

The optimal configuration of the system for dark-lesion detection (i.e., the same feature vector and classification algorithm as for bright-lesion detection) is also compared with some state-of-the-art works, in which the detection/segmentation of these lesions is performed by using classical techniques such as filtering and mathematical morphology. Table 10 summarizes the dark-lesion detection results achieved by the proposed method and by other works of the literature.

The proposed method presents comparable results with respect the representative methods involved in the comparison. In Table 10, we can see as our method achieved better results (in terms of sensitivity-specificity trade-off) than Rocha et al. [54] and Ashraf et al. [56] while the works presented in Roychowdhury et al. [55] and Junior and Welfer [57] register outperforming respect our system. In the case of Junior and Welfer [57], the presented method is only able to detect dark signs of diabetic retinopathy, i.e., an ad-hoc algorithm to identify microaneurysms and hemorrhages is developed. On contrary, in this work, a generic feature vector and classification algorithm have been proposed for describing both kind of retinal lesions: bright and dark. The work proposed in Roychowdhury et al. [55] needs a previous candidate map generation before the classification step. This kind of approach presents a high false-positive rate at pixel level and it is the main motivation of our patch-based analysis.

## 5. Conclusions

We have presented a classification procedure to identify pathological patches containing exudates, microaneurysms and hemorrhages in retinal fundus images. The discriminative texture between the healthy and pathological areas of the image is encoded in the proposed feature vector. We have seen that the combination of morphological (granulometry) and texture descriptors improve the results with respect to using only one of them. To assure the results, we used cross-validation and tested different families of classifiers.

We have seen that the classifier obtained with a particular database can be extended to be used with other databases. This fact provides to our detection system a high level of robustness and simplicity. In addition, the local analysis in which the image description and classification stages are based not only makes unnecessary the stage of segmentation or candidate generation, but it also provides an accurate location of the damaged retinal area as we have seen in Figure 11.

The proposed methodology will be the basis of high-level computer aided diagnostic algorithms under development. In particular, the output of the resulting models presented here with along additional information will be employed in the identification of the different DR stages (i.e., early, mild, moderate and severe) as well the classification between non-proliferative and proliferative DR.

## Figures and Tables

**Table 1:** AUC, accuracy, sensitivity and specificity related to the exudate detection for *E-OPHTHA exudates* database taking into account different morphological input vectors to the SVM classifier.

	Accuracy	Sensitivity	Specificity	AUC
$\gamma_{B}$	0.6405 ± 0.0572	0.6137 ± 0.1378	0.6489 ± 0.0813	0.6701 ± 0.0617
$\gamma_{L}$	0.6684 ± 0.0353	0.6009 ± 0.1449	0.6759 ± 0.0521	0.6907 ± 0.0601
$\varphi_{B}$	0.7014 ± 0.0247	0.6396 ± 0.1420	0.7090 ± 0.0426	0.7337 ± 0.0777
$\varphi_{L}$	0.5794 ± 0.0588	0.5105 ± 0.0887	0.5914 ± 0.0823	0.5783 ± 0.0366
$\gamma_{B}\varphi_{B}$	0.7554 ± 0.0161	0.6592 ± 0.1179	0.7698 ± 0.0292	0.7877 ± 0.0524
$\gamma_{B}\varphi_{L}$	0.6550 ± 0.0528	0.6223 ± 0.1243	0.6636 ± 0.0745	0.6946 ± 0.0546
$\gamma_{B}\gamma_{L}$	0.7321 ± 0.0208	0.6394 ± 0.0839	0.7465 ± 0.0271	0.7543 ± 0.0461
$\gamma_{L}\varphi_{B}$	0.7112 ± 0.0262	0.6446 ± 0.1344	0.7194 ± 0.0451	0.7482 ± 0.0684
$\gamma_{L}\varphi_{L}$	0.6685 ± 0.0320	0.5935 ± 0.1363	0.6774 ± 0.0499	0.6882 ± 0.0564
$\varphi_{B}\varphi_{L}$	0.7014 ± 0.0256	0.6346 ± 0.1376	0.7099 ± 0.0432	0.7349 ± 0.0749
$\gamma_{B}\gamma_{L}\varphi_{B}\varphi_{L}$	0.7620 ± 0.0165	0.6648 ± 0.1124	0.7762 ± 0.0300	0.7924 ± 0.0493

**Table 2:** AUC, accuracy, sensitivity and specificity related to the exudate detection for *E-OPHTHA exudates* database taking into account different input vectors to the SVM classifier. In particular, they are composed by texture and shape descriptors.

	Accuracy	Sensitivity	Specificity	AUC
LBPV	0.8205 ± 0.0320	0.7389 ± 0.1034	0.8331 ± 0.0534	0.8684 ± 0.0435
LBPV-$\varphi_{B}$	0.8369 ± 0.0336	0.7603 ± 0.0897	0.8481 ± 0.0519	0.8834 ± 0.0397
LBPV-$\gamma_{B}\varphi_{B}$	0.8445 ± 0.0276	0.7657 ± 0.0894	0.8561 ± 0.0449	0.8872 ± 0.0384
LBPV-$\gamma_{B}\gamma_{L}$	0.8447 ± 0.0228	0.7496 ± 0.1079	0.8591 ± 0.0432	0.8803 ± 0.0409
LBPV-$\gamma_{B}\gamma_{L}\varphi_{B}\varphi_{L}$	0.8533 ± 0.0245	0.7721 ± 0.0857	0.8651 ± 0.0399	0.8948 ± 0.0351

**Table 3:** AUC, accuracy, sensitivity and specificity related to the bright-lesion detection on *E-OPHTHA exudates* database for each classification method using a decision threshold δ=0.5.

	Accuracy	Sensitivity	Specificity	AUC
Random Forests	0.9508 ± 0.0084	0.4785 ± 0.1015	0.9921 ± 0.0043	0.9256 ± 0.0173
Linear-SVM	0.8533 ± 0.0245	0.7721 ± 0.0857	0.8651 ± 0.0399	0.8948 ± 0.0351
RBF-SVM	0.8796 ± 0.0229	0.8118 ± 0.0618	0.8851 ± 0.0296	0.9240 ± 0.0161
Gaussian Processes	0.8762 ± 0.0206	0.8348 ± 0.0650	0.8795 ± 0.0266	0.9353 ± 0.0174

**Table 4:** AUC, accuracy, sensitivity and specificity related to the bright-lesion detection on *E-OPHTHA exudates* database. Results were optimized to the best sensitivity/specificity trade-off.

	Accuracy	Sensitivity	Specificity	δ
Random Forests	0.8410 ± 0.0181	0.8418 ± 0.0178	0.8411 ± 0.0181	0.8999 ± 0.0214
Linear-SVM	0.8242 ± 0.0279	0.8243 ± 0.0284	0.8242 ± 0.0278	0.5771 ± 0.0958
RBF-SVM	0.8529 ± 0.0190	0.8531 ± 0.0193	0.8529 ± 0.0190	0.5835 ± 0.1012
Gaussian Processes	0.8581 ± 0.0221	0.8579 ± 0.0221	0.8579 ± 0.0222	0.5348 ± 0.0747

**Table 5:** AUC, accuracy, sensitivity and specificity related to the bright-lesion detection on *DIARETDB1* database for each classification method using a decision threshold δ=0.5.

	Accuracy	Sensitivity	Specificity	AUC
Random Forests	0.9370 ± 0.0155	0.5096 ± 0.0478	0.9889 ± 0.0056	0.8852 ± 0.0314
Linear-SVM	0.8702 ± 0.0217	0.7396 ± 0.0528	0.8849 ± 0.0260	0.8879 ± 0.0301
RBF-SVM	0.8834 ± 0.0311	0.0.7509 ± 0.0489	0.8903 ± 0.0211	0.8901 ± 0.291
Gaussian Processes	0.8703 ± 0.0189	0.7705 ± 0.0500	0.8814 ± 0.0224	0.8971 ± 0.0286

**Table 6:** AUC, accuracy, sensitivity and specificity related to the bright-lesion detection on *DIARETDB1* database. Results were optimized to the best sensitivity-specificity trade-off.

	Accuracy	Sensitivity	Specificity	δ
Random Forests	0.8037 ± 0.0319	0.8027 ± 0.0324	0.8038 ± 0.0319	0.9006 ± 0.0146
Linear-SVM	0.8112 ± 0.0316	0.8107 ± 0.0315	0.8112 ± 0.0316	0.6207 ± 0.0440
RBF-SVM	0.8166 ± 0.0322	0.8154 ± 0.0321	0.8155 ± 0.0322	0.6334 ± 0.0401
Gaussian Processes	0.8184 ± 0.0324	0.8183 ± 0.0324	0.8184 ± 0.0324	0.6023 ± 0.0518

**Table 7:** Comparison of exudate detection methods for the 47 retinal images with exudates of *DIARETDB1* database.

Methods	Sensitivity	Specificity	PPV
Sopharak et al. [21]	0.8482	0.9931	0.2548
Walter et al. [16]	0.6600	0.9864	0.1945
Welfer et al. [17]	0.7048	0.9884	0.2132
Ghafourian and Pourreza [18]	0.7828	-	-
Proposed method	0.8184 ± 0.0324	0.8183 ± 0.0324	0.4373 ± 0.1374

**Table 8:** AUC, accuracy, sensitivity and specificity related to the dark-lesion detection (microaneurysms and hemorrhages) on *DIARETDB1* database for each classification method studied in this work using a decision threshold δ=0.5.

	Accuracy	Sensitivity	Specificity	AUC
Random Forests	0.9016 ± 0.0311	0.1718 ± 0.0800	0.9818 ± 0.0035	0.8150 ± 0.0356
Linear-SVM	0.7404 ± 0.0134	0.6969 ± 0.0865	0.7462 ± 0.0245	0.7975 ± 0.0367
RBF-SVM	0.7603 ± 0.0223	0.7299 ± 0.0821	0.7576 ± 0.0311	0.8209 ± 0.344
Gaussian Processes	0.7612 ± 0.0244	0.7489 ± 0.0844	0.7630 ± 0.0350	0.8344 ± 0.0330

**Table 9:** AUC, accuracy, sensitivity and specificity related to the dark-lesion detection (microaneurysms and hemorrhages) on *DIARETDB1* database. Results were optimized to the best sensitivity-specificity trade-off.

	Accuracy	Sensitivity	Specificity	δ
Random Forests	0.7397 ± 0.0328	0.7391 ± 0.0320	0.7398 ± 0.0329	0.8645 ± 0.0329
Linear-SVM	0.7262 ± 0.0329	0.7261 ± 0.0324	0.7262 ± 0.0330	0.5170 ± 0.0591
RBF-SVM	0.7498 ± 0.0277	0.7375 ± 0.0301	0.7374 ± 0.0302	0.5554 ± 0.0601
Gaussian Processes	0.7562 ± 0.0290	0.7561 ± 0.0289	0.7562 ± 0.0290	0.5036 ± 0.0712

**Table 10:** Comparison of dark-lesion detection methods for the 45 retinal images with microaneurysms or hemorrhages of *DIARETDB1* database.

Methods	Sensitivity	Specificity	AUC
Rocha et al. [54]	0.9000	0.6000	0.7640
Roychowdhury et al. [55]	0.7550	0.9373	0.8263
Ashraf et al. [56]	0.6763	0.6278	0.6500
Junior and Welfer [57]	0.8769	0.9244	-
Proposed method	0.7561 ± 0.0301	0.7562 ± 0.0290	0.8344 ± 0.0330
